# Platelet-rich plasma may accelerate diabetic wound healing by modulating epithelial/endothelial-mesenchymal transition through inhibiting reactive oxygen species-mediated oxidative stress

**DOI:** 10.3389/fbioe.2025.1623780

**Published:** 2025-08-11

**Authors:** Youan Li, Biao Cheng, Ju Tian

**Affiliations:** ^1^ Department of Plastic Surgery, Zhongshan City People’s Hospital, Zhongshan, Guangdong, China; ^2^ Department of Plastic Surgery, General Hospital of Southern Theater Command, PLA, Guangzhou, Guangdong, China

**Keywords:** platelet-rich plasma, diabetic wound healing, oxidative stress, mitochondrial transfer, EMT/EndMT

## Abstract

The production of reactive oxygen species (ROS) and oxidative stress are central to the pathophysiology of diabetic wounds. This environment arises from the interplay of hyperglycemia, mitochondrial dysfunction, and chronic inflammation, leading to persistent damage. This hypothesis paper explores the therapeutic potential of Platelet-Rich Plasma (PRP) for accelerating diabetic wound healing. We specifically focus on PRP’s ability to modulate ROS and the key processes of Epithelial/Endothelial-to-Mesenchymal Transition (EMT/EndMT). PRP, rich in growth factors and functional platelet-derived mitochondria, shows promise in treating diabetic wounds by reducing oxidative stress and enhancing cellular processes crucial for healing. We propose that PRP accelerates healing through several interconnected mechanisms: (1) Reducing ROS production and alleviating oxidative stress; (2) Enhancing cell proliferation, migration, and angiogenesis; (3) Transferring healthy platelet-derived mitochondria to replace damaged host cell mitochondria, restoring energy metabolism; (4) Modulating cellular signaling pathways regulating ROS generation and scavenging systems, and subsequently impacts EMT/EndMT processes; and (5) Directly modulating EMT/EndMT dynamics. This hypothesis examines these proposed mechanisms and highlights future research priorities necessary to elucidate PRP’s precise mode of action and refine its clinical applications for diabetic wounds. Furthermore, the potential of PRP in treating other oxidative stress-related conditions warrants investigation.

## Introduction

Diabetes, a chronic metabolic disorder affecting over 400 million people globally, significantly reduces life expectancy and precipitates numerous complications, with impaired chronic wound healing posing a particularly challenging burden (Zhang et al., 2021). Diabetic wounds are characterized by a complex interplay of factors that disrupt the healing process, where reactive oxygen species (ROS) and oxidative stress play pivotal roles (Zhang et al., 2021). Hyperglycemia creates a hostile microenvironment that drives excessive ROS generation, disrupting the oxidant-antioxidant balance and causing oxidative stress. This impairs essential cellular functions for healing and dysregulates critical processes like epithelial/endothelial -to-mesenchymal transition (EMT/EndMT). While EMT/EndMT normally promotes cell migration and tissue remodeling, elevated ROS levels in diabetes pathologically overactivate these transitions, contributing to fibrosis and delayed healing rather than repair (S et al., 2022; Miscianinov et al., 2018). Consequently, therapeutic strategies targeting ROS modulation, angiogenesis promotion, wound microenvironment improvement, and signaling pathway regulation have become key research foci (Cai et al., 2023; Li Y. et al., 2022; Bodnár et al., 2018).

To address these challenges, Platelet-Rich Plasma (PRP) has emerged as an effective biological therapy for diabetic wound healing (Zhang and Zhao, 2024). The mechanism by which PRP promotes healing involves the release of various growth factors, such as vascular endothelial growth factor (VEGF), platelet-derived growth factor (PDGF), and transforming growth factor-beta (TGF-β). These factors are crucial for processes such as angiogenesis, collagen deposition, and the migration of repair cells to the wound site (Qian et al., 2020; Karina et al., 2019). For instance, PRP has been shown to enhance the proliferation of fibroblasts and keratinocytes, which are vital for re-epithelialization and tissue regeneration (Jafar et al., 2020). Furthermore, PRP contributes to a conducive healing environment through multifaceted mechanisms, including reducing ROS levels, enhancing cell proliferation and migration, promoting angiogenesis, and balancing inflammatory responses (Koyuncu et al., 2022). Critically, recent research reveals that functional platelet-derived mitochondria within PRP contribute substantially to healing, potentially via the transfer of these mitochondria to damaged cells to restore energy metabolism and cellular function while mitigating oxidative stress (Borcherding and Brestoff, 2023; Jin et al., 2023; Levoux et al., 2021; Boudreau et al., 2020).

Despite these established mechanisms, significant knowledge gaps persist regarding the interrelationships among PRP, ROS, and EMT/EndMT, as well as the role of PRP mitochondria in mitigating oxidative stress in diabetic wounds, improving cellular function, and cellular transdifferentiation. Therefore, this hypothesis paper aims to explore unresolved mechanisms in PRP’s action on diabetic wounds. We focus particularly on PRP’s influence on ROS-mediated EMT/EndMT pathways and its role in mitochondrial transfer. By elucidating these interactions, we seek to advance understanding and optimize PRP-based therapeutic strategies.

Building on established evidence that PRP delivers growth factors to enhance tissue repair (Zhang and Zhao, 2024; Qian et al., 2020; Karina et al., 2019) and emerging findings regarding its mitochondrial content (Borcherding and Brestoff, 2023; Jin et al., 2023; Levoux et al., 2021; Boudreau et al., 2020) and possible effects on cellular transitions such as EMT/EndMT, this proposed study introduces an integrative framework connecting these mechanisms. Although mitochondrial transfer from platelets has been documented in other settings (Jin et al., 2023; Levoux et al., 2021; Boudreau et al., 2020) and EMT/EndMT dysregulation contributes to impaired diabetic healing (S et al., 2022; Miscianinov et al., 2018), the proposed mechanism that PRP accelerates diabetic wound repair through mitochondrial transfer-mediated ROS reduction and subsequent EMT/EndMT modulation offers a distinct mechanistic advance. Consequently, it may uncover previously unrecognized connection between PRP-mediated bioenergetic recovery via mitochondrial transfer and redox-sensitive regulation of cellular plasticity (EMT/EndMT) in diabetic wounds. By delineating how mitochondrial transfer mediates ROS reduction, subsequently facilitating balanced EMT/EndMT dynamics and ultimately promoting healing, this work provides a unified mechanistic explanation for PRP’s therapeutic effects. This conceptual framework significantly extends the understanding of PRP beyond its conventional role as a growth factor source.

## Hypothesis

**FIGURE 1:**
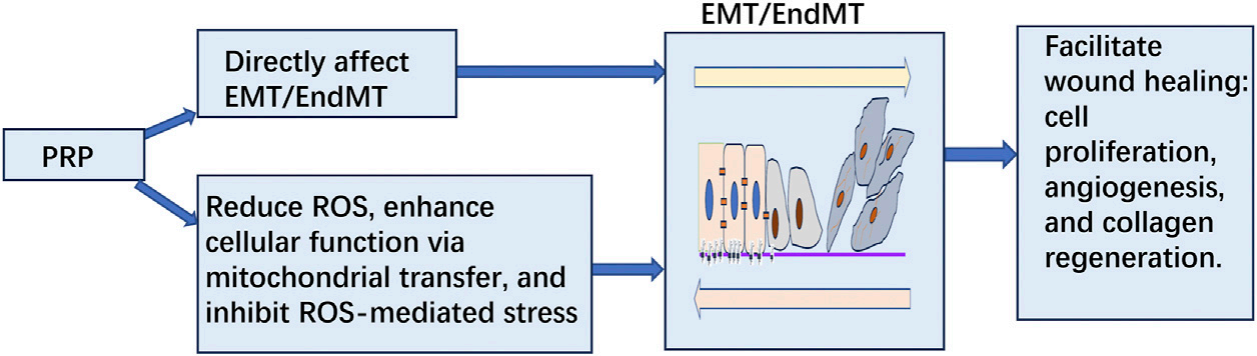
PRP may accelerate diabetic wound healing by modulating EMT/EndMT through inhibiting ROS-mediated oxidative stress.

PRP may accelerate diabetic wound healing by modulating EMT/EndMT through potentially inhibiting ROS-mediated oxidative stress (Figure 1).

The primary mechanism by which PRP promotes healing in diabetic wounds may lie in alleviating oxidative stress mediated by ROS. Specifically, upon activation, platelets within PRP release growth factors, cytokines, mitochondria, and other bioactive substances. These growth factors and other bioactive agents regulate cellular signaling pathways, influencing ROS production and clearance, and consequently lowering oxidative stress levels. Mitochondria are transferred into damaged vascular endothelial cells, epithelial cells, and fibroblasts, replacing those impaired by oxidative stress and restoring cellular energy metabolism and normal physiological functions. Together, these effects regulate the balance of EMT/EndMT. Additionally, some of the bioactive substances in PRP can directly regulate EMT/EndMT. Ultimately, these actions accelerate cellular differentiation, migration, and proliferation, expediting the healing of diabetic wounds.

PRP has exhibited considerable promise in regulating oxidative stress markers, which hold paramount importance in the pathophysiological mechanisms underlying diabetic wound healing (Fekry et al., 2025; Huang et al., 2025-01). Table1 concisely outlines the possible impacts of PRP on oxidative stress indicators (Huang et al., 2025-01; Chen et al., 2022; Josh et al., 2021; Mazloomi et al., 2025; Kargarpour et al., 2022). By targeting these oxidative stress markers, PRP addresses the fundamental issues-notably chronic oxidative stress and mitochondriial dysfunction-that contribute to delayed wound healing in diabetes. Furthermore, PRP has demonstrated potential efficacy in modulating EMT/EndMT, processes that are indispensable for effective diabetic wound healing (Table2) (Luo et al., 2025; Wang et al., 2022; Tian et al., 2024; Wang et al., 2018; Patel et al., 2017). The dysregulation of EMT/EndMT is a significant factor impairing wound healing in diabetes, and PRP may aid in restoring balance by exerting influence on crucial markers (Tian et al., 2024).

**TABLE 1:** Proposed effects of PRP on oxidative stress levels in diabetic wound healing.

Oxidative stress marker	Proposed effects of PRP	Mechanism/outcome
Malondialdehyde (MDA)	Reduction in MDA levels	PRP decreases lipid peroxidation, reducing oxidative damage to cell membranes (Fekry et al., 2025; Chen et al., 2022; Josh et al., 2021; Mazloomi et al., 2025)
NADPH Oxidase	Downregulation of NADPH oxidase activity	PRP inhibits ROS-producing enzymes, lowering overall ROS generation (Chen et al., 2022; Kargarpour et al., 2022).
ROS	Reduction in ROS levels	PRP restores mitochondrial function and enhances antioxidant defenses, reducing ROS accumulation (Huang et al., 2025-01; Chen et al., 2022).
Superoxide Dismutase (SOD)	Increase in SOD activity	PRP enhances antioxidant enzyme activity. Promoting the clearance of superoxide radicals (Fekry et al., 2025; Chen et al., 2022; Mazloomi et al., 2025).
Catalase (CAT)	Increase in catalase activity	PRP upregulates catalase, enhancing the breakdown of hydrogen peroxide into water and oxygen (Fekry et al., 2025; Mazloomi et al., 2025).
Glutathione Peroxidase (GPx)	Increase in GPx activity	PRP boosts GPx activity, reducing hydrogen peroxide and lipid hydroperoxides (Koyuncu et al., 2022).

**TABLE 2:** Proposed effects of PRP on EMT/EndMT markers in diabetic wound healing.

EMT/EndMT marker	Proposed effects of PRP	Mechanism/outcome
E-cadherin	Upregulation of E-cadherin expression	PRP promotes epithelial phenotype, enhancing cell-cell adhesion and reducing EMT progression (Luo et al., 2025; Wang et al., 2022; Tian et al., 2024).
ZO-1 (Zonula Occludens-1)	Upregulation of ZO-1 expression	PRP enhances tight junction formation, improving epithelial barrier function and reducing EMT progression (Luo et al., 2025; Wang et al., 2022; Tian et al., 2024).
Occludin	Upregulation of occludin expression	PRP strengthens cell-cell junctions, promoting epithelial integrity and wound closure (Tian et al., 2024; Wang et al., 2018; Patel et al., 2017).
Claudins	Upregulation of claudin family proteins (e.g., Claudin-1, Claudin-4)	PRP improves epithelial barrier function and reduces permeability, supporting wound healing (Luo et al., 2025; Wang et al., 2022; Tian et al., 2024).
N-cadherin	Downregulation of N-cadherin expression	PRP inhibits mesenchymal transition, supporting tissue repair and reducing fibrosis (Luo et al., 2025; Wang et al., 2022; Tian et al., 2024).
Vimentin	Downregulation of vimentin expression	PRP reduces mesenchymal marker expression, promoting epithelial stabilization (Tian et al., 2024; Wang et al., 2018; Patel et al., 2017).
Snail/Slug	Downregulation of Snail and Slug transcription factors	PRP inhibits EMT-inducing transcription factors, preventing excessive mesenchymal transition (Luo et al., 2025; Wang et al., 2022; Tian et al., 2024).
Fibronectin	Reduction in fibronectin expression	PRP decreases extracellular matrix deposition, reducing scar formation and fibrosis (Tian et al., 2024; Wang et al., 2018; Patel et al., 2017).
α-Smooth Muscle Actin (α-SMA)	Downregulation of α-SMA expression	PRP reduces myofibroblast differentiation, preventing excessive tissue contraction and fibrosis (Tian et al., 2024; Wang et al., 2018; Patel et al., 2017).

## Evaluation of the hypothesis

While oxidative stress and excessive ROS production play central roles in diabetic wound pathophysiology, ROS also function as essential signaling molecules in normal wound repair. Physiological ROS levels promote cell proliferation, differentiation, and immune defense mechanisms that are critical for proper wound healing (Huo et al., 2009). These molecules activate key pathways including nuclear factor kappa B (NF-κB) and mitogen-activated protein kinases (MAPKs), which coordinate inflammation, angiogenesis, and tissue regeneration (Huo et al., 2009; Lim et al., 2006). Chronic hyperglycemia in diabetes, however, elevates ROS production, causing mitochondrial dysfunction, compromised antioxidant defenses, and persistent oxidative stress (Caturano et al., 2025). Such dysregulation impairs the healing cascade, resulting in delayed wound closure and defective tissue repair. PRP may help rebalance ROS signaling by reducing oxidative damage while maintaining ROS-mediated physiological functions. Clarifying these mechanisms could inform therapeutic strategies to manage oxidative stress in diabetic wounds. Although promising, this hypothesis warrants rigorous evaluation against existing evidence.

### The potential impact of PRP on the ROS-EMT/EndMT axis in diabetic wound healing

**FIGURE 2:**
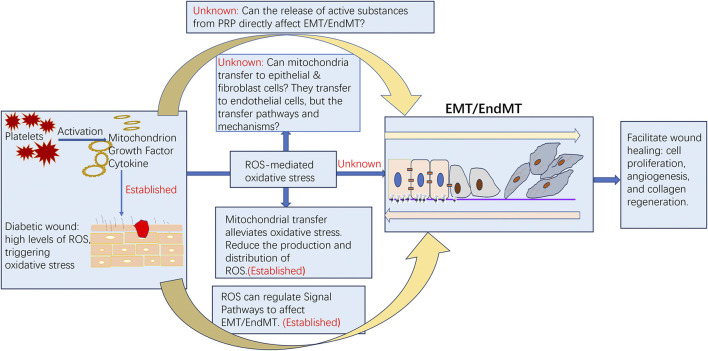
Current research advances and future exploration directions of PRP, ROS, oxidative stress, and EMT/EndMT in the healing process of diabetic wounds.

The absence of proof connecting PRP to EMT/EndMT regulation is acknowledged (Figure 2). The role of PRP in diabetic wound healing has garnered significant attention due to its potential to regulate various biological processes, including the ROS and EMT/EndMT axis. Diabetic wounds are characterized by delayed healing, often attributed to oxidative stress, impaired angiogenesis, and dysfunctional cellular responses. PRP, abundant in growth factors and cytokines, offers therapeutic benefits by addressing these underlying issues. It has been demonstrated that PRP can reduce oxidative stress by enhancing the antioxidant capacity of the wound environment, potentially mitigating the adverse effects of ROS on wound healing (Zhang C. et al., 2019). In the context of diabetic wounds, the dysregulation of EMT and EndMT hinders effective healing. PRP may influence the ROS-EMT/EndMT axis by modulating Wnt/β-catenin, PI3K/Akt, MAPK, NF-κB, and TGF-β signaling pathways (Table 3) (Zhang C. et al., 2019; Ebrahim et al., 2021; Liu et al., 2019; Zhang L. et al., 2023; Ju et al., 2023; Guo et al., 2022; Liu et al., 2024; Fang et al., 2019), promoting a more conducive healing environment. For instance, PRP has been shown to activate the PI3K/Akt pathway, which plays a crucial role in cell survival and proliferation. This activation can counteract the harmful effects of ROS, thereby supporting the cellular functions necessary for effective wound healing (Ebrahim et al., 2021). By addressing oxidative stress and regulating the EMT/EndMT axis, PRP can enhance cellular responses, angiogenesis, and tissue regeneration in diabetic wounds (Zhang C. et al., 2019; Ebrahim et al., 2021; Fang et al., 2024; Karas et al., 2024). However, further research is needed to elucidate the precise mechanisms by which PRP affects these pathways and to optimize its application in clinical settings.

**TABLE 3:** Mechanistic analysis of PRP’s modulation of key signaling pathways in diabetic wound healing.

Pathway	Mechanism of PRP modulation	Biological effects
Notch (Zhang et al., 2019a; Ebrahim et al., 2021)	PRP activates Notch receptors and downstream target	Enhances tissue regeneration, angiogenesis, and cell proliferation while regulating inflammation and fibrosis
Wnt/β-Catenin (Liu et al., 2019)	PRP stabilizes β-catenin, enhancing its nuclear translocation and transcriptional activity.	Promotes epithelial cell survival, proliferation, and re-epithelialization
PI3K/Akt (Zhang et al., 2023a)	PRP activates PI3K/Akt signaling, enhancing cell survival and antioxidant enzyme expression.	Reduces oxidative stress, prevents apoptosis, and supports angiogenesis.
MAPK (ERK, JNK, p38) (Guo et al., 2022)	PRP activates ERK and inhibits JNK/p38 pathways.	Enhances cell proliferation and migration while reducing inflammation and oxidative stress.
NF-κB (Liu et al., 2024)	PRP inhibits NF-κB activation, reducing pro-inflammatory cytokine	Balances inflammatory responses and reduces oxidative damage.
TGF-β (Fang et al., 2019)	PRP modulates TGF-β signaling, regulating EMT/EndMT processes.	Promotes tissue regeneration while inhibiting excessive fibrosis.

### The role of platelet-derived mitochondria in diabetic wound healing within PRP

The role of platelet mitochondria derived from PRP in diabetic wound healing presents a novel treatment paradigm (Figure 2). Mitochondrial transfer generally refers to the movement and exchange of mitochondria between different cells or organelles, potentially playing a significant role in cellular injury repair and energy metabolism. After transfer, the mitochondria integrate into the mitochondrial network of recipient cells through fusion and fission. Jin et al. (2023) demonstrated that platelets serve as important donors of mitochondria, and platelet-derived mitochondria can promote wound healing by reducing cell apoptosis in vascular endothelial cells caused by oxidative stress. They also proposed that ultrasound is a more suitable method for activating platelets to release respiratory mitochondria. Additionally, these mitochondria released by platelets can transfer to mesenchymal stem cells, enhancing their angiogenic capacity through metabolic reprogramming (Levoux et al., 2021). Platelet-derived mitochondria can also bind to neutrophils, promoting enhanced rolling adhesion of neutrophils to the vascular wall and facilitating the activation and migration of inflammatory cells, thereby regulating inflammatory responses and wound healing (Boudreau et al., 2020). These findings further expand our understanding of platelet function and provide new insights into the role of platelet-derived mitochondria in wound healing. However, the specific mechanisms underlying platelet mitochondrial transfer and how transferred mitochondria improve cell function remain to be further explored in this study. Therefore, transferring healthy mitochondria to replace those damaged by oxidative stress in wound cells (including but not limited to mesenchymal stem cells, neutrophils, vascular endothelial cells, epithelial cells, and fibroblasts) represents a potential novel therapeutic approach for chronic diabetic wounds.

### The direct impact of PRP on the EMT/EndMT process

While PRP has been shown to have antioxidant properties and to reduce oxidative stress in some studies (Koyuncu et al., 2022; Chen et al., 2022; Josh et al., 2021; Mazloomi et al., 2025; Kargarpour et al., 2022), the direct link between PRP’s ability to modulate EMT/EndMT and its antioxidant effects on ROS-mediated oxidative stress in diabetic wound healing is not well-established (Figure 2). To contextualize this, EMT and EndMT are fundamental processes in tissue repair. During normal wound healing, partial EMT allows epithelial and endothelial cells to adopt a more mesenchymal phenotype, enhancing their migratory capacity to cover the wound bed and initiate angiogenesis (S et al., 2022; Tian et al., 2024; Simon et al., 2014; Gutmann et al., 2021; Lambert and Weinberg, 2021). Conversely, EndMT contributes to the formation of activated fibroblasts and myofibroblasts crucial for extracellular matrix deposition and wound contraction (Miscianinov et al., 2018; Tian et al., 2024). However, in the pathological milieu of diabetic wounds, chronic hyperglycemia and sustained oxidative stress drive excessive and dysregulated EMT/EndMT (S et al., 2022; Miscianinov et al., 2018). This pathological transition promotes fibroblast -to-myofibroblast conversion, excessive collagen deposition, vascular rarefaction, and ultimately fibrosis, which impedes re-epithelialization and functional tissue regeneration (S et al., 2022; Miscianinov et al., 2018; Patel et al., 2017). Thus, therapeutic modulation of EMT/EndMT aims not for complete inhibition, but for restoring its precise spatiotemporal dynamics–promoting beneficial cell migration and angiogenesis while suppressing persistent activation that fuels fibrosis. PRP has been shown to regulate various cellular pathways such as Wnt/β-catenin, MAPK, and NF-κB (Liu et al., 2019; Zhang L. et al., 2023; Ju et al., 2023; Fang et al., 2019), which are key modulators of EMT/EndMT. Additionally, PRP contains a multitude of activators (e.g., TGF-β, PDGF) that can directly influence the EMT/EndMT process (Simon et al., 2014; Gutmann et al., 2021; Lambert and Weinberg, 2021). Based on these observations, it is plausible to infer that PRP may have a potential impact on the EMT/EndMT process, potentially shifting the balance away from the pathological, fibrotic transition observed in diabetes towards a more controlled, healing-promoting transition. For instance, certain growth factors within PRP may inhibit excessive EMT/EndMT activation by modulating related signaling pathways (e.g., counteracting sustained TGF-β/Smad signaling), while others might transiently support necessary mesenchymal characteristics for cell migration.

In summary, while the hypothesis that PRP may accelerate diabetic wound healing by modulating EMT/EndMT through inhibiting ROS-mediated oxidative stress is plausible, it requires further investigation and validation through rigorous scientific research. Studies that specifically investigate the mechanisms by which PRP affects EMT/EndMT and its antioxidant effects on ROS-mediated oxidative stress in diabetic wounds would be valuable in advancing our understanding of this complex process.

## Testing the hypothesis

To test the hypothesis, researchers would conduct comprehensive cellular, animal experiments, and clinical trials (Table 4), examining the impact of PRP on ROS levels in diabetic wounds and assess its potential to reduce oxidative stress, evaluating the modulation of EMT/EndMT markers in wound tissues by PRP, investigating the signaling pathways involved in PRP-mediated regulation of EMT/EndMT and wound healing, assessing functional outcomes such as wound size reduction, re-epithelialization, and granulation tissue formation in diabetic animals treated with PRP, and comparing its efficacy with standard wound care therapies to determine if PRP offers additional benefits in accelerating diabetic wound healing.

**TABLE 4:** PRP based wound healing experimental framework.

Phase	Stage	Details
Phase 1: Preclinical Mechanistic Validation	*In Vitro* Studies	-High-glucose cell models → PRP treatment → Mitochondrial transfer tracking → ROS/EMT marker analysis.
	Animal Studies	- Diabetic rodent models → PRP ± inhibitors → Wound healing metrics → Histopathology. - Compare PRP with standard therapies.
Phase 2: Translational Biomarker Discovery	Multi-Omics Profiling	- Integrate transcriptomic, proteomic, and metabolomic data → Identify predictive biomarkers.
	*Ex Vivo* Human Tissue Validation	- Test the efficacy of PRP on wound tissues taken from diabetic patients→ Confirm conserved mechanisms (ROS/EMT modulation).
Phase 3: Clinical Pilot Studies	Phase I/II Trials	- Population: Diabetic patients with chronic ulcers. Interventions: PRP vs placebo/standard care → Stratify by baseline oxidative stress/EMT marker levels.
Phase 4: Long-Term Efficacy & Mechanism Refinement	Mechanistic Refinement	- Monitor long-term outcomes in PRP treated-patients.

### *In vitro* experiments designed to investigate the impact of PRP on ROS-mediated oxidative stress and the EMT/EndMT axis

Researchers can create a well-established diabetic rat model (such as those already available in the research field, like the streptozotocin - induced diabetic rat model) and isolate wound - related cells (including endothelial cells, keratinocytes, and fibroblasts) from appropriate biological samples. Typically, the establishment of the diabetic rat model is part of animal experiments. These cells are then cultured in a high-glucose medium to simulate the diabetic environment. Meanwhile, PRP is extracted and prepared from the blood of non-diabetic rats, with its growth factor and mitochondrial content enhanced through ultrasonic treatment. The cells are divided into experimental groups (treated with PRP alone, ROS inhibitors such as N-acetylcysteine, and EMT inhibitors such as EMT inhibitor-1 and EMT inhibitor-3), and a control group (untreated). Subsequently, the migration, proliferation, and invasion capabilities of the cells in each group are evaluated, and the levels of ROS and the expression of EMT/EndMT-related markers are detected. Additionally, the expression levels of target genes and the activity of specific signaling pathways are analyzed, while single-cell sequencing technology is employed to explore changes in the gene expression profile before and after treatment. Existing studies show PRP alleviates oxidative stress in tendon (Tognoloni et al., 2023), intervertebral disc (Lian et al., 2024), and human umbilical vein endothelial cells (HUVECs) (Wei et al., 2024a). In tendon cells, it boosts antioxidant enzyme expression via Nrf2 (Tognoloni et al., 2023). In intervertebral disc cells, it lowers ROS and enhances GPX4 activity (Lian et al., 2024). In high-glucose-damaged HUVECs, it restores angiogenesis, cuts ROS by 47%, and reduces inflammatory cytokines (Wei et al., 2024a).

### In animal experiments, the impact of PRP on ROS-mediated oxidative stress and the EMT/EndMT axis can be investigated

Diabetic rat ulcer models are divided into four groups: diabetic control group, PRP treatment group, ROS inhibitor treatment group, and EMT inhibitor treatment group. Regular wound healing assessments (area, time, appearance) and histopathological exams (angiogenesis via CD31/CD34 staining, collagen neoformation via Masson’s/Sirius Red) are conducted. ROS-related proteins and genes are analyzed by Western blot and RT-qPCR. EMT markers (epithelial, endothelial, mesenchymal, and others) are detected by Western blot, immunocytochemistry and immunohistochemistry. Blood glucose, oxidative stress indicators (SOD, GSH-Px, MDA), and inflammatory cytokines (TNF-α, IL-6, IL-1β) are measured. Statistical analysis compares differences across groups and time points, providing insights into wound healing, inflammation, antioxidant status, and EMT processes. Animal experiments have demonstrated that PRP can mitigate lipid peroxidation and ROS accumulation by inhibiting ferroptosis (Zhou et al., 2024). Moreover, PRP promotes angiogenesis through the VEGFA/VEGFR2/ERK signaling pathway (Wei et al., 2024b), while concurrently suppressing high glucose-induced endothelial cell dysfunction and mesenchymal transition (Wei et al., 2024a).

### In cellular and animal experiments, the research focuses on the impact of mitochondrial transfer on vascular endothelial cells, keratinocytes, and fibroblasts

Existing research has revealed that in conditions such as diabetes, hypertension, or ischemic diseases, mitochondrial transfer can reverse mitochondrial fission and oxidative stress in endothelial cells (Li G. et al., 2022; Ajoolabady et al., 2024), while also inhibit the abnormal proliferation of keratinocytes and fibroblasts (such as in keloid formation) (Zhang M. et al., 2023). Based on this, an experimental study can be designed to investigate the transfer of mitochondria from platelets to vascular endothelial cells, keratinocytes, and fibroblasts. Vascular endothelial cells, keratinocytes, and fibroblasts can be isolated from animals with diabetic ulcers. Mitochondrial transfer from platelets to cells damaged by diabetic conditions can be examined through coculture experiments. Cells will be cultured in high-glucose medium to simulate diabetic conditions. Fluorescently labeled platelet mitochondria will be cocultured with these cells (experimental group) and with normal glucose-treated cells (control group) to assess mitochondrial transfer. Fluorescence microscopy and confocal microscopy will be used to track intracellular mitochondrial distribution and quantify transfer efficiency at multiple time points.

To evaluate the impact of platelet mitochondrial transfer on oxidative stress and cellular function, four experimental groups will be established: the healthy control group will comprise animals administered normal glucose treatment to establish baseline parameters; the diabetic ulcer model group will include animals subjected to high-glucose treatment to induce ulcerative pathology; in the functional platelet treatment group, diabetic ulcer models will be administered intact platelets to assess mitochondrial transfer efficacy; and the respiration-inhibited platelet treatment group will consist of diabetic ulcer models treated with platelets pre-inhibited by mitochondrial respiration inhibitors to investigate mechanistic dependencies. Skin ulcer tissues will be systematically collected from all groups for integrated transcriptomic and metabolomic sequencing, enabling the identification of differentially expressed redox-related genes and metabolite profiles. These multi-omics analyses will elucidate how platelet mitochondria modulate oxidative stress pathways and facilitate tissue functional recovery, as evidenced by quantitative indicators including wound healing dynamics, mitochondrial membrane potential stability, and antioxidant enzyme activities.

## Implications of the hypothesis

The hypothesis presents a novel mechanism for PRP’s therapeutic action in diabetic wound healing, extending beyond its established role as a growth factor delivery system to include oxidative stress regulation and EMT/EndMT modulation. This expanded understanding of PRP’s multifaceted healing properties provides new insights into optimizing diabetic wound treatment. The involvement of EMT/EndMT is particularly significant, as these processes govern cell migration, proliferation, and differentiation during tissue repair—suggesting PRP could strategically manipulate these transitions to accelerate healing. The emphasis on ROS-mediated oxidative stress inhibition further underscores PRP’s potential to restore redox balance, a critical factor impaired in diabetic wounds that often hinders cellular function and tissue regeneration. The proposed mitochondrial transfer mechanism offers an additional dimension, where PRP may restore cellular energy metabolism by replenishing damaged mitochondria in metabolically compromised wound environments. Finally, PRP’s direct regulation of EMT/EndMT balance could prove instrumental in preventing fibrosis while promoting healthy tissue regeneration, addressing a key complication in chronic wound management. Collectively, these mechanisms position PRP as a multifunctional therapeutic approach with potential applications extending beyond diabetic wound healing to other oxidative stress-related pathologies.

## Discussion

PRP has demonstrated therapeutic potential for diabetic wounds, primarily due to its high concentration of growth factors essential for tissue repair (Alamdari et al., 2021; Azam et al., 2024; Karmakar et al., 2024; Wang et al., 2022-01; Orban et al. 2022; Saad et al., 2022). A clinical trial reported that diabetic patients treated with PRP demonstrated significantly improved healing rates compared to the control group (Orban et al. 2022), while long-term follow-up data revealed reduced infection rates and lower amputation risks (Saad et al., 2022). A systematic review further substantiates these findings, with meta-analytic data showing PRP increases wound healing likelihood and decreases amputation risk (Saad et al., 2022).

The therapeutic mechanisms of PRP have been implicated to extend beyond established effects on inflammation, angiogenesis, and extracellular matrix remodeling (Ebrahim et al., 2021; Wang et al., 2023; Xu et al., 2020; Lian et al., 2014). Emerging evidence suggests PRP may regulate oxidative stress and influence EMT/EndMT transitions through mitochondrial transfer and ROS scavenging. This polypharmacological approach simultaneously activates multiple regenerative pathways while addressing both oxidative damage and its underlying cause—mitochondrial dysfunction.

While synthetic biomaterials like photothermal hydrogels (Wang et al., 2024) or ATP-responsive prodrug systems (Qi et al., 2024) target specific wound aspects, PRP offers inherent biological complexity without synthetic limitations. However, key questions remain regarding PRP’s precise regulation of EMT/EndMT transitions and ROS modulation. PRP likely influences EMT/EndMT through multiple pathways, including the regulation of oxidative stress via ROS scavenging and mitochondrial transfer, as well as the activation of pro-regenerative signaling pathways such as PI3K/Akt and TGF-β. These actions collectively help balance EMT/EndMT transitions, promoting tissue repair while preventing excessive fibrosis or epithelial dysfunction.

Standardization challenges—including centrifugation protocols, platelet concentration, and leukocyte content—significantly affect therapeutic outcomes (Fadadu et al., 2019; Zhang N. et al., 2019; Popescu et al., 2021). Double-spin centrifugation and calcium chloride activation, for instance, alter growth factor release kinetics and platelet yields. Moreover, it is crucial to acknowledge potential adverse effects associated with PRP therapy. Reported adverse effects include postoperative infections, inflammation, nodule development, allergic reactions, and even blindness, with postoperative infections being the most frequently documented (Arita and Tobita, 2024). Furthermore, long-term safety concerns warrant attention, especially for diabetic patients who face elevated baseline risks for malignancies. The pro-angiogenic properties of growth factors in PRP raise theoretical concerns about potentially exacerbating cancer risk in this population. Therefore, in addition to optimizing PRP formulations, comprehensive evaluation of the therapy’s efficacy and risks is essential. Establishing robust post-treatment follow-up protocols to monitor for potential adverse effects is equally critical.

## Conclusion

Collectively, this hypothesis suggests that PRP may act as a multifunctional regulator of diabetic wound pathogenesis by coordinating mitochondrial transfer, ROS-EMT/EndMT modulation, and redox homeostasis within a cohesive therapeutic mechanism. PRP may accelerate healing through a distinct mechanism that targets mitochondrial dysfunction, oxidative stress, and aberrant cellular transitions, surpassing conventional growth factor therapies. Functional platelet-derived mitochondria directly correct metabolic deficits in diabetic wounds, while coordinated suppression of ROS overproduction and precise EMT/EndMT modulation prevents fibrotic progression and restores regenerative capacity. The synergistic effects of mitochondrial bioenergetic recovery, oxidative stress mitigation, and cellular transition regulation collectively counteract the pathological environment perpetuating chronic diabetic wounds.​​ While these findings expand our understanding of PRP’s action mechanisms, further studies must precisely characterize its molecular targets and optimize clinical protocols. Additional research should also explore PRP’s applicability to other oxidative stress-mediated pathologies.

## Data Availability

The original contributions presented in the study are included in the article/supplementary material, further inquiries can be directed to the corresponding authors.
